# Determination of single cryoablation outcome within 30 to 60 seconds of freezing based on ice impedance

**DOI:** 10.1111/jce.14097

**Published:** 2019-08-19

**Authors:** Boaz Avitall, Ken S. Lizama, Arthur Kalinski, Nicolas Coulombe, Timothy G. Laske

**Affiliations:** ^1^ Division of Cardiology University of Illinois at Chicago Chicago Illinois; ^2^ Medtronic Montreal Quebec Canada; ^3^ Medtronic Saint Paul Minnesota

**Keywords:** atrial fibrillation, cryoballoon ablation, impedance, pulmonary vein isolation

## Abstract

**Background:**

A direct indicator of effective pulmonary vein isolation (PVI) based on early ice formation is presently lacking.

**Objective:**

The initial impedance rise within 30 to 60 seconds (sec) of single cryoablation relating to ice on the distal surface of the cryoballoon could; predict effective PVI with early termination, the need for prolonging the cryoablation, or failure to achieve effective ablation.

**Methods:**

Impedance measurements were taken between two ring electrodes, at the anterior balloon surface and at the shaft behind the balloon. Ice covering the anterior ring leads to impedance rise. Single cryoablation (eight animals, 37 veins) was applied for 90 to 180 sec. Cryoapplication was terminated if the impedance reached ≥500 Ω. Impedance levels at ≤60 sec of cryoablation were divided into three groups based on the characteristics of the impedance rise. PVI was confirmed acutely and at 45 ± 9 days recovery by electrophysiology mapping and histopathology.

**Results:**

At 60 sec of freezing, an impedance rise of 34.1 ± 15.2 Ω (13‐50 Ω) and slope of the impedance rise (measured during 15‐30 sec of cryoapplication) less than 1 Ω/sec resulted in failed PVI. An impedance rise of 104.4 ± 31.5 Ω (76‐159 Ω) and slope of 2 Ω/sec resulted in 100% PVIs. An impedance rise of 130.9 ± 137.8 Ω (40‐590 Ω) and slope of 10 Ω/sec resulted in 100% PVIs with early termination at 90 sec.

**Conclusion:**

The efficacy of single cryoablation can be defined within 30 to 60 sec based on ice impedance. Three unique impedance profiles described in this investigation are associated with the uniformity and thickness of the ice buildup on the anterior surface of the balloon. One cryoablation with an adequate impedance rise is needed for successful outcomes.

## 1 INTRODUCTION

**Figure 1:**
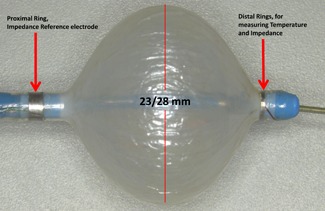
Cryoballoon instrumented with a distal ring electrode positioned 2 mm from the balloon and a proximal reference ring electrode. The impedance was measured between the two electrodes. Accumulation of ice on the balloon anterior surface expands to cover the anterior ring electrode causing the impedance to increase rapidly once the ring is totally covered

The cryoballoon has become a common device for the treatment of paroxysmal atrial fibrillation.^1^ With the introduction of the Arctic Front Advance balloon, the freeze time has evolved to minimize extracardiac damage while maintaining clinical efficacy. Recently, Avitall et al^2^ have examined the relationship between ice formation on the anterior surface of the balloon and electrical impedance changes. The impedance was measured between a ring electrode positioned on the 23‐mm cryoballoon catheter shaft 2 mm from the anterior surface of the balloon and a reference ring electrode positioned on the shaft behind the balloon (Figure 1). It was concluded that the impedance rise of 500 Ω at ≤90 seconds (sec) with freeze time of 90 sec resulted in 100% pulmonary vein isolation (PVI). Using these electrodes, the increasing impedance is a direct measure of the ice thickness covering the anterior ring electrode. It provides real‐time feedback on the quality of the ablation and defines the cryoapplication termination time based on ice formation, limiting ice expansion to extracardiac tissues. While time to PV electrical isolation algorithms is now widely used to titrate the cryoapplication time it requires consistent PV potentials. Aryana et al^3^ reported that 81% of PVs sleeve potentials where recorded forcing the clinician to apply prolong freeze times to these veins. Furthermore, despite utilizing the time to isolation still, some of the cryoapplications fail to achieve long‐term PV isolation.^3^, ^4^, ^5^, ^6^ PV reconnection may reflect tissue stunning as a result of uneven ice formation.^7^ The early innovation of the cryoballoon envisioned this technology will effectively isolate the PVs without the need for intracardiac recordings. By introducing direct biophysical measures that reflect ice thickness brings this technology a bit closer to this reality.

This investigation sought to define whether the initial impedance rise within 30 to 60 sec of single cryoablation could; predict effective PVI with early termination, the need for prolonging the cryoablation, or failure to achieve effective ablation independent of time to isolation.

## 2 METHODS

All procedures were performed in compliance with the American Heart Association's for animal research and approved by the Animal Care Committee of the University of Illinois at Chicago.

As shown in Figure 1, an electrode ring was placed on the shaft 2 mm from the anterior surface of the 23‐mm Arctic Front Advance balloon. An additional electrode was placed proximally behind the balloon and was used as the reference electrode for the impedance measurements. The rings were connected to a current source (Model 6221 DC and AC Current Source; Keithley) and a multimeter (Model 2100 6 ½ Digit Multimeter; Keithley) while continuous measurements were taken with an add‐on program through Microsoft Office Excel. This allowed for the impedance continuously to be measured before, during, and after the cryoapplication had been initiated. In addition, the intraballoon temperature during the freezing and thawing was recorded by the CryoConsole (Medtronic, Minneapolis, MN).

Eight canines (30‐35 kg) underwent anesthesia induction with intravenous propofol (0.5 mg/kg) and maintenance with inhaled isoflurane 1.00% to 1.75% via endotracheal intubation. Intra‐arterial blood pressure and esophageal temperature monitoring were performed throughout the entire experimental protocol, and core temperature was maintained at 38.0 ± 1.3°C using a heating blanket.

A 20‐pole deflectable catheter was inserted into the external jugular vein and positioned in the high right atrium extending into the coronary sinus as a reference. Transseptal puncture was performed, and a 14F deflectable sheath was inserted into the left atrium (LA). The chamber anatomy, PV orifices, and LA voltage maps were generated using a 20‐pole deflectable catheter and the EnSite NavX 3D (St Jude Medical, St Paul, MN) navigation system. PV sleeve conduction and voltage maps were assessed preintervention acutely postcryoapplication and at the terminal study 45 ± 9 days of recovery. We have made every effort to record PV sleeve electrical activity during the cryoapplication; however, given the size of the PV and the short PV muscle sleeve, it was not consistently possible. Consequently, this investigation examined whether the characteristics of impedance changes during the first minute of freezing can be the sole parameter by which the cryoapplication is titrated. A 0.035′′ guidewire was advanced into the vein, and the cryoballoon instrumented with the ring electrodes was inserted and positioned to engage the PVs.

As previously described, the cryoballoon was inflated, and PV occlusion was assessed and every effort was made to create a good PV occlusion using a cold saline technique.^8^ To summarize, the cold saline technique quantifies balloon‐tissue contact and provides an assessment of leaks surrounding the balloon without the use of fluoroscopy and radiopaque contrast. Cryoballoons instrumented with an anterior thermocouple can detect the temperature decrease associated with an injection of 5 cc of 4°C saline solution while the balloon is positioned to occlude the PV. The cold saline injection results in characteristic temperature changes that correlate with the quality of the occlusion. PVI success was validated in every vein immediately postablation and 6 weeks of recovery by the electrical mapping, and histopathologic analysis of the PVs ostia as well as the intraballoon freeze and thaw characteristics.

### 2.1 Data analysis

The data consisted of a continuous recording of the impedance and the balloon internal temperature as provided by the CryoConsole. The PVs were divided into three distinct groups based on the characteristic of the impedance rise at the first 60 sec of cryoapplication.

Type I impedance defined as early and exponential impedance increase, type II, and type III are slow and linear increase throughout the cryoapplication distinguished by their slope. The cryoapplication was terminated if the impedance exceeded 500 Ω but not until at least 90 sec of ablation. The data were analyzed at 30, 40, 50, and 60 sec of cryoapplication. The final impedance data for each of the impedance types, the nadir temperature and the thawing times were analyzed as well.

PV isolation was defined by pre‐ and postablation and following 6 weeks of recovery using three‐dimensional electrical activity and conduction block and confirmed by tissue analysis using tetrazolium chloride and multisectional trichrome stain histological sections.

### 2.2 Statistics

**Figure 2:**
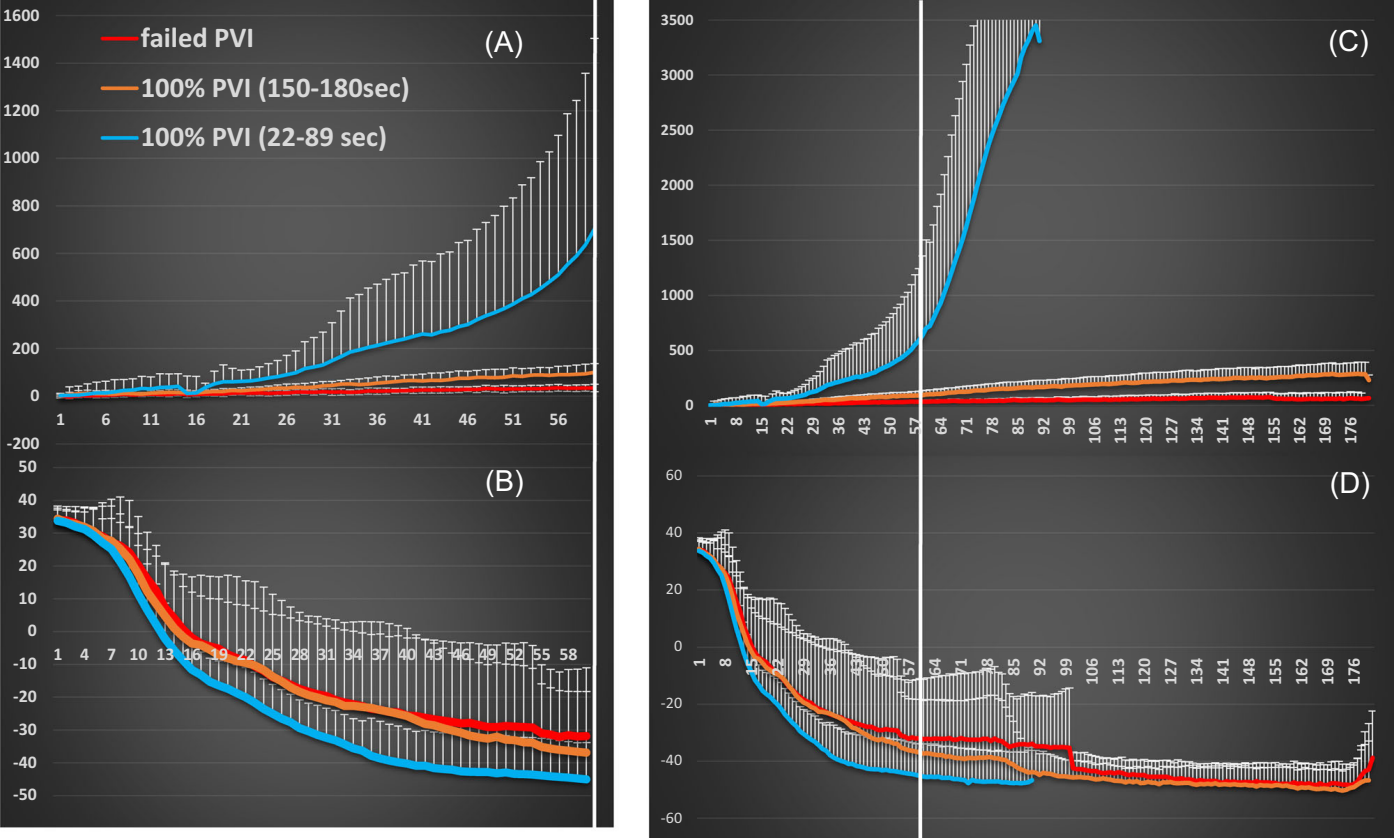
Impedance (A, C) and cryoballoon internal temperature (B, D) that resulted in successful and failed PVIs. Rapid impedance rise (blue curves are shown in A and C) represent all the successful cryoapplications that were terminated at 90 seconds. Significant separation is already noted at 30 seconds of freezing. Slow impedance rise resulting in successful PVI and applied for 152 to 180 seconds is shown in orange. Failed PVI with minimal impedance rise is shown in red. PVI, pulmonary vein isolation

Nonparametric Kruskal‐Wallis and Mann‐Whitney test were used to determine statistical differences between each of the three unique characteristics of impedance types as shown in Figure 2. Similarly, the same analysis was performed on the console temperature and thaw times. All data are expressed as a mean ± SD. A *P* ≤ .05 was deemed significant. IBM SPSS Statistics 24 software was used for all statistical computations.

## 3 RESULTS

**Figure 3:**
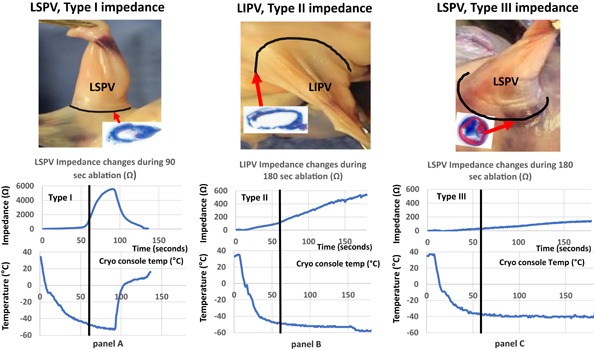
Tetrazolium stained gross pathology of pulmonary veins 4 weeks postcryoablation, the trichrome stained histological section taken from the base of the PVs (upper panels). The ice impedance and internal balloon temperatures during the cryoapplication for the three types of impedance increases shown in the lower panels. LIPV, left inferior pulmonary vein; LSPV, left superior pulmonary vein; PV, pulmonary vein

As shown in Table 1 and Figures 2 and 3, within 60 sec after the initiation of the cryoapplication, the impedance measured from the ring electrodes indicates the likelihood of successful ablation. At 30 sec into the cryoapplication type I impedance is significantly increased vs type II and III impedance and the balloon temperature is also significantly lower in the successful vs the failed ablations. However, as the ablation progress, the internal balloon temperatures plateaus and type II vs III balloon temperature increase are no longer significantly different. In contrast, the rate of impedance increase in the successful lesions significantly outpaces the impedance change in the PVs whose cryoisolation failed (Tables 1 and 2 and Figure 2). An additional differentiation parameter is the rate of impedance increase as shown in Table 2. The slope of the impedance rise was measured starting at 15 sec (once the cryoballoon has reached maximal liquid nitrous oxide flow) up to 30 or 60 sec of the freeze. Type I impedance rate of increase is significantly greater than type II, and type II is significantly greater than type III (*P* < .005). The rate of impedance increase is not different if the slope is measured between 15 to 30 and 15 to 60 sec of cryoapplication. In contrast to the slope of the impedance increase, the internal balloon rate of temperature drop does not follow the change in impedance and none of the values are significantly different between the three impedance types.

**Table 1:** The change (Δ) of ice impedance and the cryoballoon internal temperature (displayed by the console) during the first minute of cryoablation for the three types of impedance profiles

	∆ In ice impedance (Ω)				Internal balloon temperature (°C)			
	30 s	40 s	50 s	60 s	30 s	40 s	50 s	60 s
Type I (N¦=¦16) 100% PVI (90¦s)	130.9¦±¦137.8 (range, 39.8‐589.5)*, **, ^a^, ^b^	251.2¦±¦299.8 (range, 55.0‐1118.9)*, **, ^a^, ^b^	368.2¦±¦429.5 (range, 62.9‐1733.4)*, **, ^a^, ^b^	701.5¦±¦802.1 (range, 118.9‐3329.7)*, **, ^a^, ^b^	−35.4¦±¦9.0 (range, −19 to −53)**, ****, ^a^, ^b^	−43.4¦±¦9.8 (range, −30 to −72)**, ****, ^a^, ^b^	−44.3¦±¦11.1 (range, −32 to −74)****, ^a^, ****, ^b^	−49.0¦±¦9.5 (range, −30 to −74)**, ***, ^a^, ^b^	
Type II (N = 13) 100% PVI (152‐180 s)	44.7 ± 15.3 (range, 22.9‐74.9)****, ^c^	65.7 ± 23.8 (range, 34.3‐100.4)***, ^c^	81.5 ± 31.4 (range, 22.2‐121.4)***, ^c^	104.4 ± 31.5 (range, 75.9‐158.8)***, ^c^	−31.9 ± 7.1 (range, −17 to −45)**, ^c^	−38.1 ± 6.1 (range, −31 to −52)***, ^c^	−41.1 ± 6.3 (range, −32 to −55)****, ^c^	−43.1 ± 6.7 (range, −33 to −56)****, ^c^
Type III (N = 8) failed PVI (180 s)	18.8 ± 10.0 (range, 7.2‐32.4)	24.3 ± 11.1 (range, 12.8‐37.9)	29.5 ± 14.6 (range, 8.2‐45.7)	34.1 ± 15.2 (range, 13.4‐50.2)	−25.6 ± 4.2 (range, −20 to −32)	−29.4 ± 6.4 (range, −19 to −38)	−38.4 ± 5.0 (range, −34 to −47)	−38.4 ± 5.7 (range, −35 to −49)

*Note*: Average ± SD (range).

Abbreviations: NS, not significant; PVI, pulmonary vein isolation.

* *P* ≤ .005.

** *P* ≤ .02.

*** *P* ≤ .05.

**** *P* = NS.

^a^ Type I vs II.

^b^ Type I vs III.

^c^ Type II vs III.

**Table 2:** The rate of impedance (Ω/s) and temperature (°C/s) change measured by the slope for the three types of impedance at 15‐30 and 15‐60 s of freeze

	Average impedance slope, mean (Ω/s)		Average temperature slope, mean (°C/s)	
	15‐30 s	15‐60 s	15‐30 s	15‐60 s
Type I	9.9 ± 13.4^a^, *, ^b^, *	11.8 ± 16.2^a^, *, ^b^, *	−1.5 ± 0.4^a^, ***, ^b^, ***	−0.7 ± 0.2^a^, ***, ^b^, ***
Type II	2.0 ± 1.0^c^, *	1.9 ± 0.8^c^, *	−1.2 ± 1.0^c^, ***	−0.8 ± 0.5^c^, ***
Type III	0.7 ± 0.3	0.6 ± 0.2	−1.5 ± 0.3	−0.7 ± 0.1

*Note*: Average ± SD (range).

Abbreviation: NS, not significant.

^a^ Type I vs II.

^b^ Type I vs III.

^c^ Type II vs III.

* *P* ≤ .005.

** *P* ≤ .05.

*** *P* = NS.

An example of three types of impedance changes, the internal balloon temperature and the corresponding PV and histology is shown in Figure 3.

A total of 29/37 (78%) of the cryoapplications has resulted in successful PV isolation and had an impedance that was ≥76 Ω at 60 sec of cryoapplication. All of the failed ablations had an impedance of ≤50 Ω (8 of 37, 22%). Unsuccessful ablations were typically documented at the right superior pulmonary vein (RSPV) (five of eight lesions) and left superior pulmonary vein (three of eight lesions). The reason for the ablation failure is related to the anatomical characteristics of the RSPV. The RSPV is the largest diameter (14.1 ± 1.7 mm) vein and is subdivided into three branches, as a result, the balloon distal shaft is typically anchored at the superior branch of the vein resulting in off‐center position and poor balloon anterior surface approximation with the PV ostia. As shown in Figure 2, ablations with an impedance that exceeded 500 Ω at 90 sec are characterized by an exponential rise in impedance, at times rising to a few thousand ohms at 90 sec (type I impedance rise). A slower linear rise in impedance (type II), reaching 92 to 543 Ω and the cryofreeze time was 152 to 180 sec. In two of these ablations, the change in impedance exceeded 500 Ω at 152 and 166 sec. All the veins demonstrating type I and II impedance rise at 30 to 60 sec of the initiation of the cryoapplication were isolated acutely after ablation and at the terminal study 6 weeks later. In contrast, all the cryoapplications that demonstrated 13 to 50.2 Ω (type III impedance rise) at 60 sec of cryoapplication resulted in PVI failure acutely and at the terminal study 6 weeks later.

Furthermore, the electrical mapping was confirmed by tissue tetrazolium chloride staining and trichrome histological analysis.

The maximal impedance change, the internal balloon nadir temperature, and thawing times for the successful and unsuccessful cryoapplications are shown in Table 3, Figure 2C,D, and Figure 3. The rate and extent of the temperature drop during type I impedance rise is consistent with good PV occlusion and rapid and thicker ice formation as depicted by the lower nadir temperature and slower thawing time (Table 3).

**Table 3:** The maximal impedance change, the internal balloon nadir temperature and thawing times for the successful and unsuccessful cryoapplications

	Max Δ imp (Ω)	Nadir 0°C	Thaw time to 0°C, s	Thaw time to 15°C, s	Total thaw time, s
Type I (N = 16) 100% PVI (90 s)	3368.9 ± 1876.3 (range, 596.1‐5577.4)^a^, ^b^, *, **	−55.13 ± 9.4 (range, −33 to −76)^a^, ^b^, **, ****	12.8 ± 7.3 (range, 4‐36)^a^, ^b^, ***, ****	34.1 ± 13.1 (range, 7‐56)^a^, ^b^, **, ****	41.9 ± 13.7 (range, 18‐66) ^a^, ^b^, ***, ****
Type II (N = 13) 100% PVI (152‐180 s)	291.3 ± 113.2 (range, 92.1‐542.5)^c^, **	−49.7 ± 8.4 (range, −36 to −64)^c^, **	8.9 ± 4.1 (range, 5‐17)^c^, ****	27.9 ± 10.9 (range, 15‐50) ^c^, ****	33.5 ± 11.2 (range, 22‐52)^c^, ****
Type III (N = 8) failed PVI (180 s)	75.1 ± 40.2 (range, 24.4‐142.7)	−40.9 ± 6.7 (range, −32 to −51)	7.1 ± 3.1 (range, 4‐12)	22.8 ± 7.7 (range, 15‐34)	27.1 ± 9.1 (range, 16‐40)

*Note*: Average ± SD (range).

Abbreviations: imp, impedance; NS, not significant; PVI, pulmonary vein isolation.

^a^ Type I vs II.

^b^ Type I vs III.

^c^ Type II vs III.

* *P* ≤ .005.

** *P* ≤ .02.

*** *P* ≤ .05.

**** *P* = NS.

With type II impedance rise, the internal balloon temperature at 30, 40, 50, and 60 sec is not significantly different from the failed ablations. Similarly, the 15 sec and total thawing time between type II vs type III impedance rise are not different providing additional evidence that the early impedance change is a superior indicator of cryoablation success or failure. An example of the three types of impedance rise, the corresponding internal balloon temperature, and the consequent lesion is shown in Figure 3. In all the three examples the internal balloon temperature dropped below −40°C and the failed ablation (panel C) would be considered adequate.

Anatomically and by histology, no differences in the characteristics and the extent of the PV ostial lesion was noted between type I and type II impedance rise. Whereas the cryoapplication that resulted in type III impedance rise resulted in smaller and segmental PV lesions.

## 4 DISCUSSION

The characteristic of the impedance rise at 30 to 60 sec of the cryoapplication is an effective indicator of the ablation outcome. Successful PVI can be achieved with 90 sec of cryoablation if at 30 sec of cryoablation the impedance is ≥40 Ω (mean, 130.9 ± 137.8 Ω) and ≥500 Ω at 90 sec. An impedance of less than 40 Ω at 30 sec but ≥76 Ω at 60 sec (mean, 104.4 ± 31.5 Ω) will require to prolong cryoapplication up to 180 sec if the impedance is less than 500 Ω. The cryoapplication was also terminated once the impedance reached 500 Ω at less than 180 sec. Low impedance rise (≤50 Ω; mean, 34.1 ± 15.2 Ω) at 60 sec will not achieve PVI and is an indication for balloon repositioning.

Each of the groups described has a unique impedance profile that is associated with the uniformity and thickness of the ice buildup on the anterior surface of the balloon. The impedance rise provides significantly greater discrimination of a failed vs successful cryoapplication compared with the internal balloon temperature profile.

A recent publication examined the clinical efficacy of the cryoballoon PVI in an effort to titrate the cryoablation dosing to achieve maximal efficacy. The method used is based on time to PVI plus 120 sec.^9^ Presently, many practitioners use one to two 180 sec applications. For permanent PVI to be achieved, there must be a circumferential ice formation and tissue temperature lower than −20°C.^10^ The addition of 120 sec is an effort to ensure a successful outcome. For each individual PV, effective cryoablation varies and it is a function of the PV occlusion, balloon PV position, surface area of the balloon‐tissue contact, atrial blood flow to mention a few.^11^ A direct measure of ice formation would be much more precise in guiding the cryoapplication and define the ablation efficacy.

The conductive properties of ice have been well described in biological tissues.^12^, ^13^, ^14^, ^15^ In our initial publication on this subject, we have reported once the impedance level has reached ≥500 Ω in ≤90 sec of freeze time, the cryoapplication can be terminated at 90 sec with 100% likelihood of achieving sustained PVI.^2^

In this investigation, we have examined the early sequence of impedance rise, particularly between 30 to 60 sec of cryoapplication to provide the clinician with an early indicator of efficacy with a single cryofreeze. In addition, we characterized the unique features of cryo impedance associated with 90 sec successful PVI (type I impedance), ≤180 sec successful PVI (type II impedance), and PVI failure (type III impedance).

**Figure 4:**
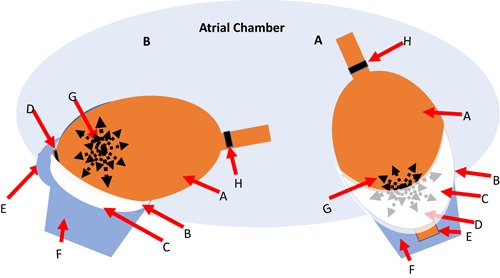
Graphic representation of the cryoballoon positions into the PV and ice formation impacting the cryo impedance. An important variable that impacts the ice formation on the anterior balloon surface and durable PVI is the cryoballoon PV occlusion and its position in relation to the PV ostia. As illustrated in panel A, the ideal position is when the balloon perpendicular to the PV ostia with the balloon anterior surface is in good uniform contact with the tissues. The liquid nitrous oxide spray in the Arctic Front Advance is positioned as such that the anterior surface has the maximal heat exchange. Given the LA and PV anatomy, at times the balloon engages the PV in a shallow angle (panel B) exposing part of the balloon anterior surface to the atrial blood flow. Depending on the extent the angular position the ice formation will be uneven and slow to cover the anterior ring electrode if at all. In such a condition the impedance rise is slow reflecting the uneven distribution of ice around the balloon and the ring electrode, and as a result, increasing likelihood of failure to create a circumferential lesion. LA, left atrium; PV, pulmonary vein; PVI, pulmonary vein isolation A. Cryoballoon B. PV ostia C. Ice formation on the balloon exposed to the LA blood flow D. Anterior ring electrode position E. Cryoballoon shaft. F. PV G. Cryo spray position at the distal half of the balloon H. Proximal ring electrode

The rate of ice formation and consequently the impedance rise on the Arctic Front Advance is determined by multiple factors associated with the balloon cooling power and external thermal loads. The most important of these factors is the cessation of blood flow over the balloon from the PV and the extent of heat exchange defined by the anterior surface area in contact with the targeted tissues. An important variable is the cryoballoon position in relation to the PV ostia. As illustrated in Figure 4A, the ideal position is when the balloon perpendicular to the PV ostia and the balloon anterior surface is in good uniform contact with the tissues. The liquid nitrous oxide spray in the Arctic Front Advance is positioned such that the anterior surface has the maximal heat exchange. Given the LA and PV anatomy, at times the balloon engages the PV in a shallow angle (Figure 4B) exposing part of the balloon anterior surface to the atrial blood flow. Depending on the extent the angular position the ice formation will be uneven and slow to cover the anterior ring electrode if at all. In such a condition the impedance rise is slow reflecting the uneven distribution of ice around the balloon and the ring electrode, and as a result, increasing likelihood of failure to create a circumferential lesion (Figure 4).

In this investigation, we have defined three types of impedance profiles that reflect the balloon ice formation. Type I is comprised of two distinct slopes, an early rise, and a rapid exponential second rise. The early rise reflects the time needed for the ring electrode to be covered by the expanding ice on the anterior surface of the balloon and once that occurs the rate of temperature decrease and ice formation is reflected by the very rapid increase in impedance. Type II is defined by a slow gradual and linear impedance rise that is as a result of the angular position of the balloon in the PV ostia. Inadequate and uneven ice formation may lead to PV isolation failure regardless of a good PV occlusion. A slow impedance rise indicates ice formation requiring a longer time to grow around the balloon surface covering the anterior ring electrode in an uneven fashion. Finally, a very low (≤50 Ω) or no impedance rise (type III) reflects partial or no circumferential ice formation on the ring electrode suggesting shallow balloon position within the vein, poor contact, and/or partial PV occlusion, and as a result PV isolation failure as shown in Figure 4.

The internal balloon temperature changes during freezing and extent of the temperature drop during type I impedance changes (exponential impedance increase) is consistent with good PV occlusion and longitudinal position of the balloon at the PV ostia resulting in rapid and thicker ice formation as depicted by the slower thawing time (Table 3). With type II impedance rise (slower linear rise) the early internal balloon temperature decreases at 30, 40, and 60 sec, which is not significantly different from the failed ablations (type II vs type III). The 15 sec and total thawing time between type I and type III are significantly different, whereas type II vs type III are not different providing additional evidence that the early impedance change is a superior indicator of cryoablation success or failure.

Successful cryotissue ablation not only requires ice to form but such ice formation needs to create intracellular irreversible damage as previously described.^14^ In addition in our initial publication, we have noted that ablations that were terminated once 500 Ω was reached in ≥90 sec, resulted in tissue recovery. Consequently, in this investigation, we have maintained the 90 sec as a minimal ablation time. Impedance rise of ≥500 Ω before 90 sec of freeze time, 10 to 20 sec prolongation of the cryoapplication leads to several thousand ohms impedance rises. Careful analysis of the extracardiac tissues did not document phrenic, esophageal, or lung damage.^2^

In this preclinical assessment, we have determined for the first time that within the first 30 to 60 sec of cryoapplication it is possible to define the quality of the cryofreeze and the time needed to achieve durable PVI based on the early rise in ice impedance. In the human PVs and LA are larger and the 28 mm cryoballoon is used, these differences are likely to result in changes of the rate and extent of ice formation. However, the fundamental relationship of the impedance rise, ice thickness, and PVI success should remain. The ultimate utility of this technique will need to be defined in a human clinical study.

As of now, time‐to‐PVI remains the most powerful proven predictor of acute and long‐term PVI durability as demonstrated by several published studies. However, PV sleeve potential is not always possible to record. Furthermore, while cryoapplication time to isolation allows titration of the cryoapplication PV reconnection are reported and likely a major contributing factor in AF recurrence.^3^, ^4^, ^5^, ^6^ The early determination of durable PVI using the cryoballoon technology will simplify the procedure and likely improve AF ablation success.

### 4.1 Limitations

The study is a proof of concept performed in an animal model. Human clinical trials will be needed. The balloon instrumented by the ring electrode is a prototype based on the Arctic Front Advance catheter design. The impedance ranges and the time to cryo termination based on the impedance levels may need further fine‐tuning in the human. However, the ablation method used in this investigation establishes initial parameters for the application of this concept in human cryoballoon PVI procedures. The cryo ice impedance would not be useful in large common PV ostia where PV occlusion is not possible. Furthermore, at the initial phase of this investigation, we made an effort to record the PV potentials in an effort to correlate the impedance rise and the timing to PVI; however, using the standard Arctic Front Advance balloon instrumented with an Achieve catheter, we could not consistently record the PV muscle sleeve potentials because of the short PV muscle sleeve of the canine model.

We recognize the fact that the number of cryoapplications analyzed in this investigation is small and it is a result of the present sensitivities and limitations placed on large animal research especially with regard to the canine model. While the porcine model is more “acceptable” model and commonly used the anatomical differences compared with the human heart are very significant.^16^

## 5 CONCLUSION

In this preclinical study, the efficacy of single cryoablation can be defined within 30 to 60 sec of cryoapplication based on the rate of ice impedance increase. Low impedance rise (≤50 Ω; mean, 34.1 ± 15.2 Ω) at 60 sec or poor rate of impedance rise measured between 15 and 30 sec of the cryoapplication (<1 Ω/sec) will not achieve PVI and is an indication for balloon repositioning. Successful cryoablation is defined by achieving ≥76 Ω at 60 sec (mean, 104.4 ± 31.5 Ω) or slow but persistent rate of impedance rise (2 Ω/sec) and will require to prolong cryoapplication of up to 180 sec if the impedance is less than 500 Ω. A successful PVI can be achieved with 90 sec of cryoablation if the impedance is ≥40 Ω (mean, 130.9 ± 137.8 Ω) at 30 sec and ≥500 Ω at 90 sec of cryoablation. In addition, the rapid rate of impedance rise (10 Ω/sec) within 15 to 30 sec of the cryofreeze is an indication that the cryoapplication can be terminated at 90 sec. The three unique impedance profiles described in this investigation are associated with the uniformity and thickness of the ice buildup on the anterior surface of the balloon that can be defined within 30 to 60 sec of cryoapplication. In this animal study type I and type II impedance profiles are 100% predictive of durable PVI while type III impedance profile is 100% predictive of PVI failure. Only one cryoapplication with an adequate impedance rise is needed for successful PVI.

## CONFLICT OF INTERESTS

BA receives research funding and is a consultant to Medtronic.
